# Supplementary figures and images for: Automatic estimation of heading date of paddy rice using deep learning
Source: Plant Methods. 2019 Jul 13;15:76. doi: 10.1186/s13007-019-0457-1 (PMC6626381; doi:10.1186/s13007-019-0457-1)

**a**

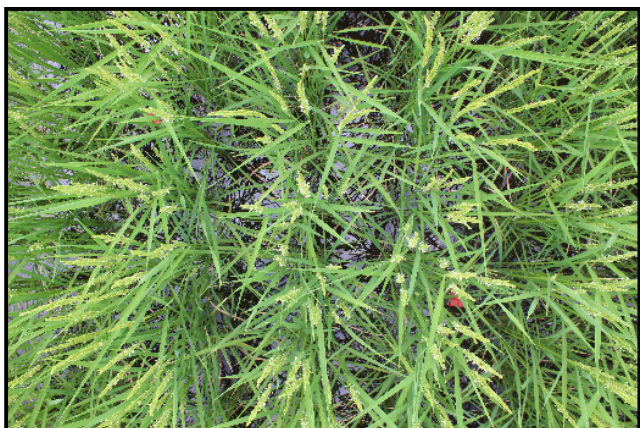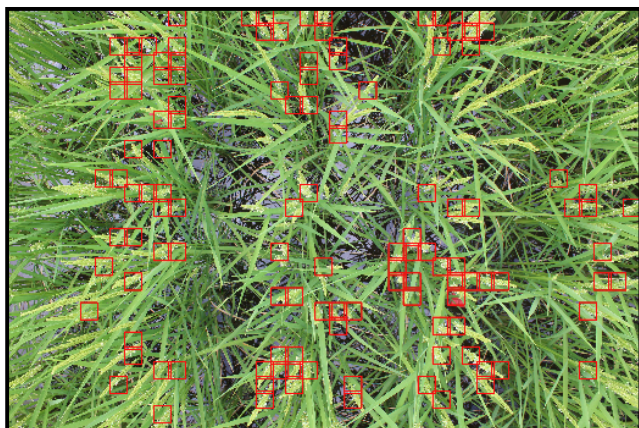

**b**

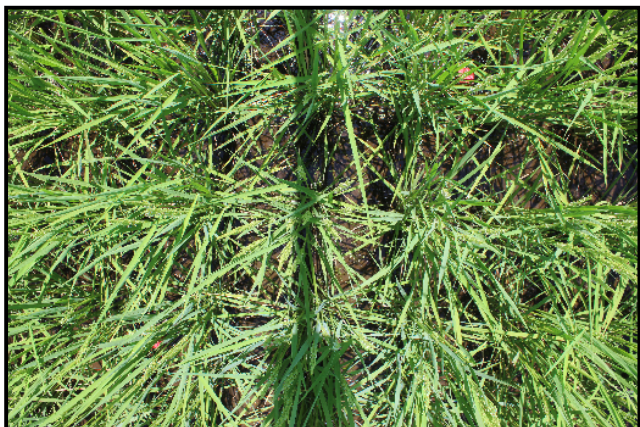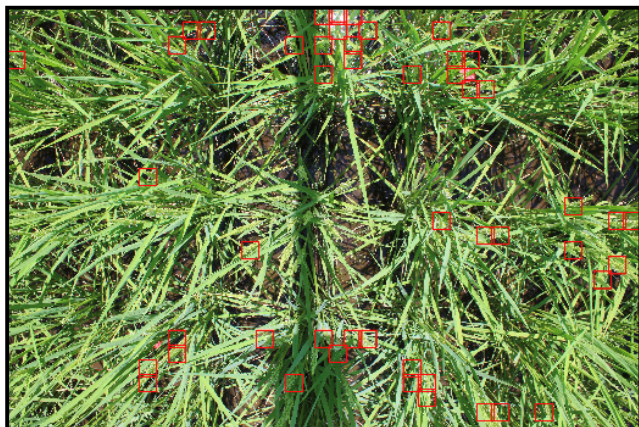

**c**

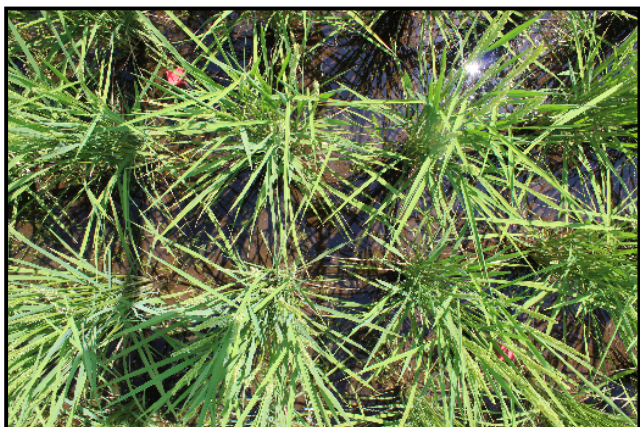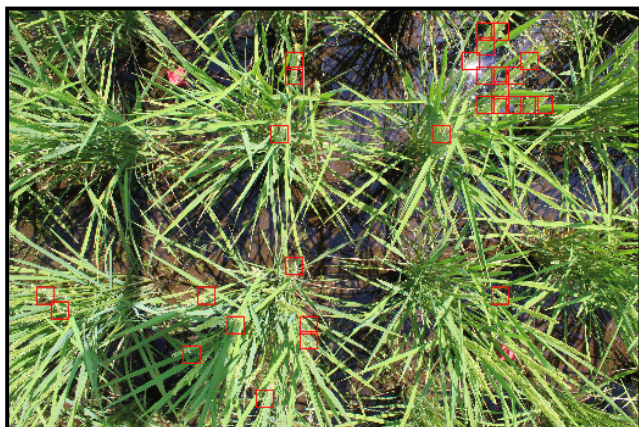

Supplement: Supplementary file 1 — Additional file 1. Flowering Region Detection in Koshihikari. It is a figure depicting flowering region detection in crop images of Koshihikari-1, Koshihikari-2 and Koshihikari-3. [file 13007_2019_457_MOESM1_ESM.pdf]

50% flowering in Koshihikari-1

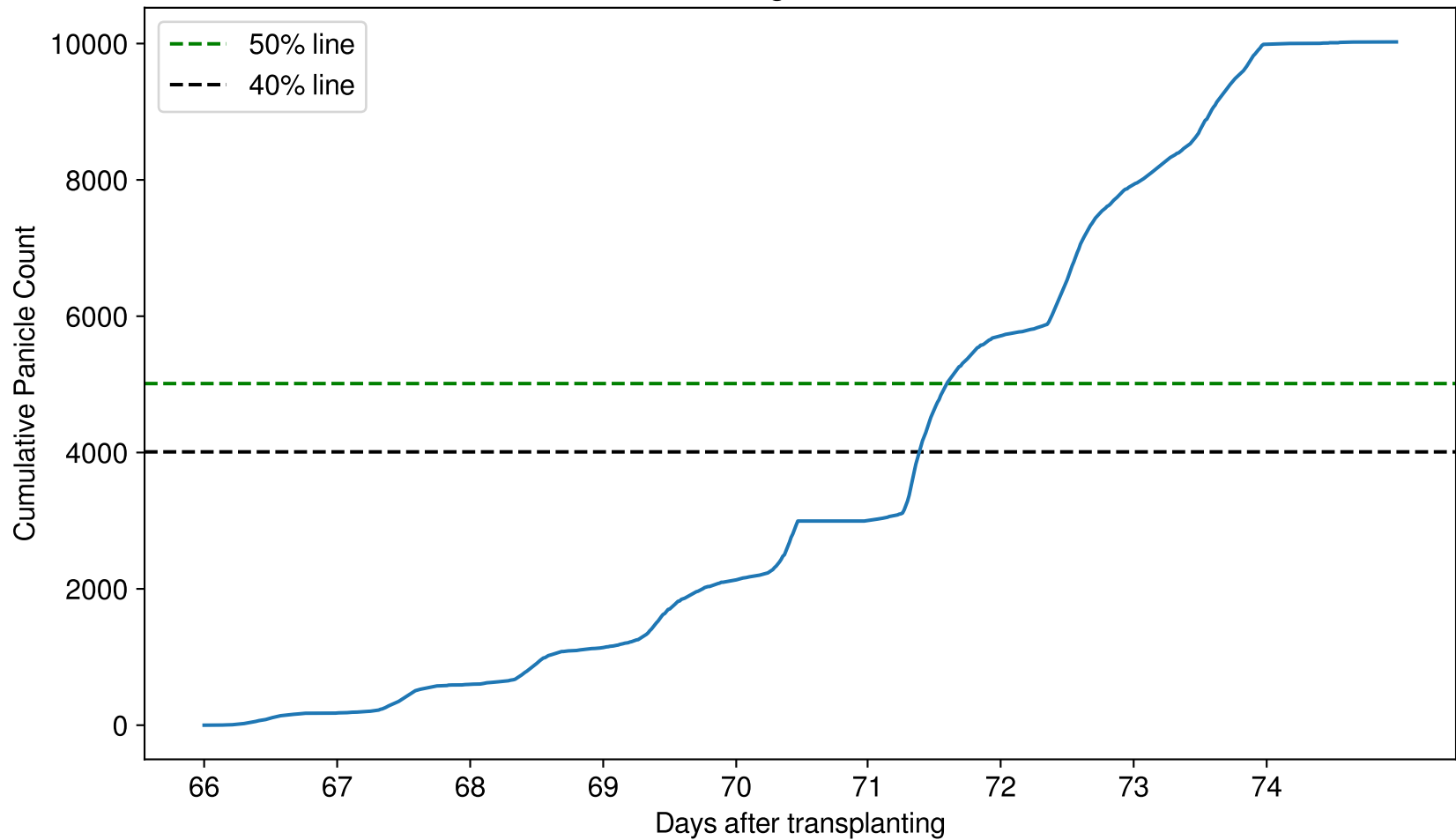

50% flowering in Koshihikari-2

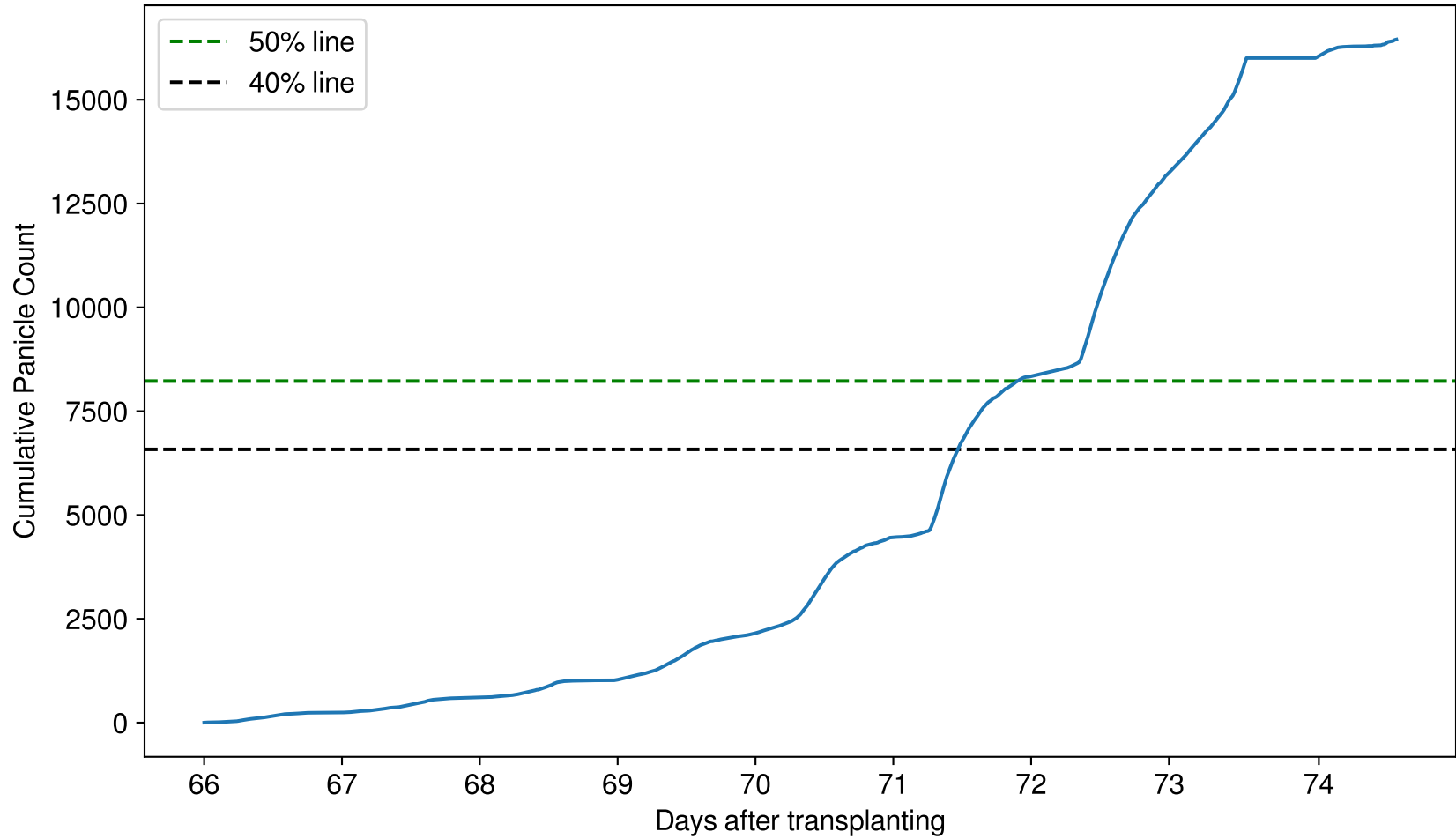

50% flowering in Koshihikari-3

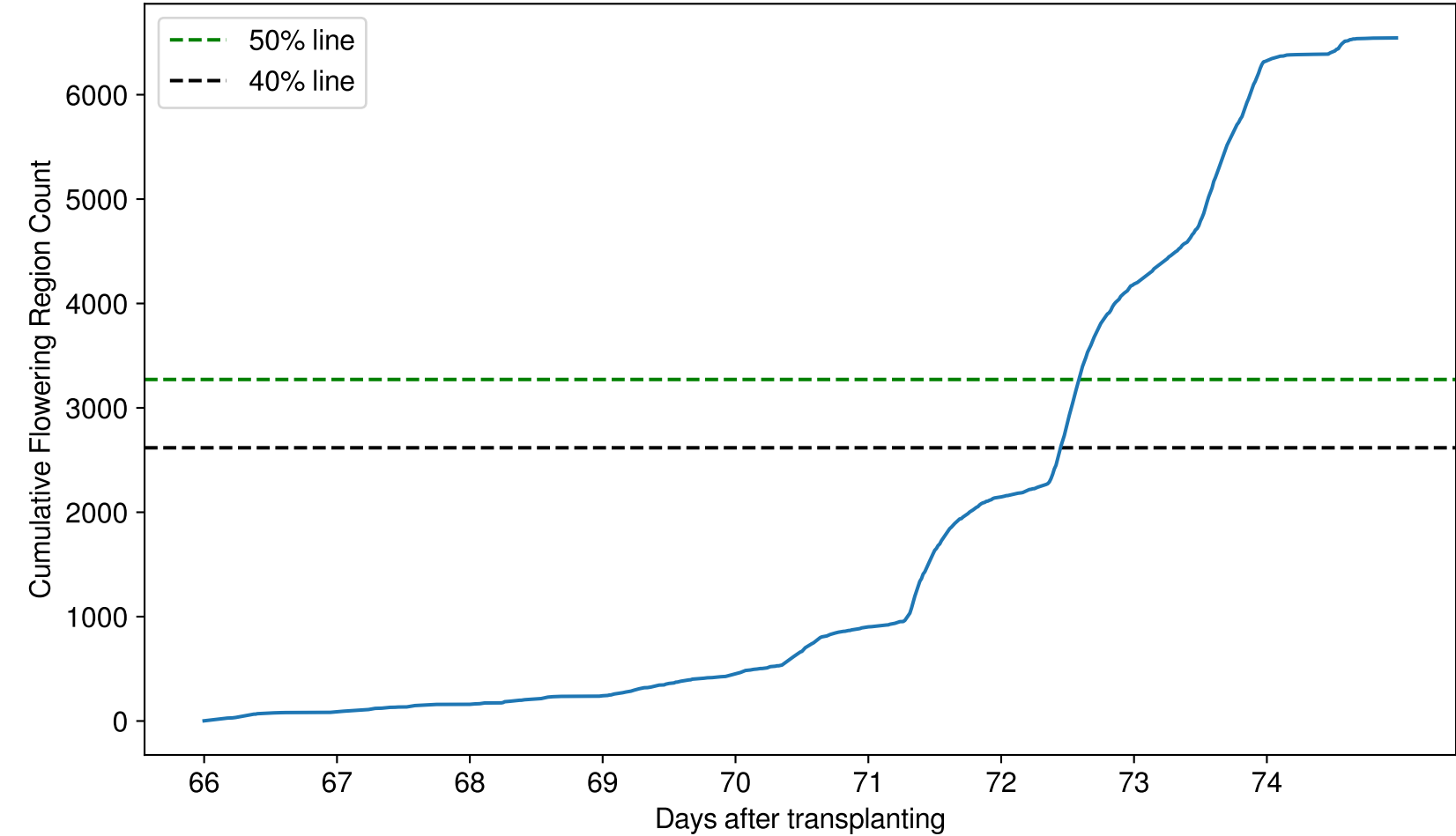

Supplement: Supplementary file 2 — Additional file 2. Flowering Stage Graphs for Koshihikari. It contains graphs depicting observed 50% flowering stage using crop images in Koshihikari-1, Koshihikari-2 and Koshihikari-3. [file 13007_2019_457_MOESM2_ESM.pdf]
